# NTCP deficiency in mice protects against obesity and hepatosteatosis

**DOI:** 10.1172/jci.insight.127197

**Published:** 2019-07-25

**Authors:** Joanne M. Donkers, Sander Kooijman, Davor Slijepcevic, Roni F. Kunst, Reinout L.P. Roscam Abbing, Lizette Haazen, Dirk R. de Waart, Johannes H.M. Levels, Kristina Schoonjans, Patrick C.N. Rensen, Ronald P.J. Oude Elferink, Stan F.J. van de Graaf

**Affiliations:** 1 Tytgat Institute for Liver and Intestinal Research, Amsterdam University Medical Centers, University of Amsterdam, Amsterdam, Netherlands.; 2 Department of Medicine, Division of Endocrinology, and Einthoven Laboratory for Vascular and Regenerative Medicine, Leiden University Medical Center, Leiden, Netherlands.; 3 Department of Experimental Vascular Medicine, Amsterdam University Medical Centers, University of Amsterdam, Amsterdam, Netherlands.; 4 Laboratory of Metabolic Signaling, École Polytechnique Fédérale de Lausanne*,* Lausanne, Switzerland.; 5 Department of Gastroenterology and Hepatology, Amsterdam Gastroenterology and Metabolism, Amsterdam University Medical Centers, University of Amsterdam, Amsterdam, Netherlands.

**Keywords:** Hepatology, Metabolism, Obesity, Transport

## Abstract

Bile acids play a major role in the regulation of lipid and energy metabolism. Here we propose the hepatic bile acid uptake transporter Na^+^ taurocholate cotransporting polypeptide (NTCP) as a target to prolong postprandial bile acid elevations in plasma. Reducing hepatic clearance of bile acids from plasma by genetic deletion of NTCP moderately increased plasma bile acid levels, reduced diet-induced obesity, attenuated hepatic steatosis, and lowered plasma cholesterol levels. NTCP and G protein–coupled bile acid receptor–double KO (TGR5–double KO) mice were equally protected against diet-induced obesity as NTCP–single KO mice. NTCP-KO mice displayed decreased intestinal fat absorption and a trend toward higher fecal energy output. Furthermore, NTCP deficiency was associated with an increased uncoupled respiration in brown adipose tissue, leading to increased energy expenditure. We conclude that targeting NTCP-mediated bile acid uptake can be a novel approach to treat obesity and obesity-related hepatosteatosis by simultaneously dampening intestinal fat absorption and increasing energy expenditure.

## Abstract 2

Targeting bile acid uptake simultaneously dampens intestinal fat absorption and increases energy expenditure, suggesting a potential approach to treat obesity and obesity-related hepatosteatosis.

## Introduction

Bile acids, well-known for their pivotal role in dietary fat absorption, are increasingly recognized as complex and important hormonal contributors to many metabolic pathways (1–3). Bile acids can activate a range of membrane and nuclear receptors, which are abundantly expressed inside and outside the enterohepatic system. Activation of, for example, the farnesoid X receptor and the G protein–coupled bile acid receptor (GPBAR1 or TGR5) by (synthetic) receptor-specific agonists has been shown to decrease body weight, improve glucose tolerance, enhance energy expenditure, and reduce inflammation in mice (4–7). This indicates that targeting bile acid signaling can be beneficial, but the simultaneous appearance of side effects teaches us that chronically targeting a single receptor is not ideal (8–10). Continuous bile acid receptor activation differs from the physiologic situation in which bile acid dynamics follow a meal-dependent rhythm (11). Therefore, we aimed to temporarily stimulate bile acid signaling and hypothesized that inhibition of hepatic bile acid uptake would induce metabolic benefits via a limited extension of the postprandial time frame in which endogenous bile acids signal to peripheral tissues.

In the liver, bile acids are synthesized from cholesterol and follow an efficient cycle of intestinal and hepatic (re)uptake (12). The Na^+^ taurocholate cotransporting polypeptide (NTCP, *SLC10A1*), exclusively expressed in the liver (13), plays a central role in this enterohepatic circulation of bile acids as the main uptake transporter of conjugated bile acids from the (portal) blood into the liver, which was recently demonstrated in vivo using NTCP-KO mice (13) and in individuals with NTCP gene mutations (14, 15). NTCP-KO mice display a delay in plasma clearance of conjugated bile acids, thereby creating a transient systemic overflow of these bile acids (13, 16). We used this mouse model to investigate the metabolic effect of prolonged bile acid signaling in a diet-induced obesity setting.

Here, we show that NTCP deficiency increased postprandial plasma bile acids, which led to a TGR5-independent reduction in body weight, less hepatic steatosis, and lower serum cholesterol. Therefore, partial inhibition of hepatic clearance of bile acids from portal and systemic blood can be used as a novel strategy to treat obesity and obesity-related hepatosteatosis.

## Results

### Absence of NTCP prevents body weight gain and hepatic steatosis.

**Figure 1:**
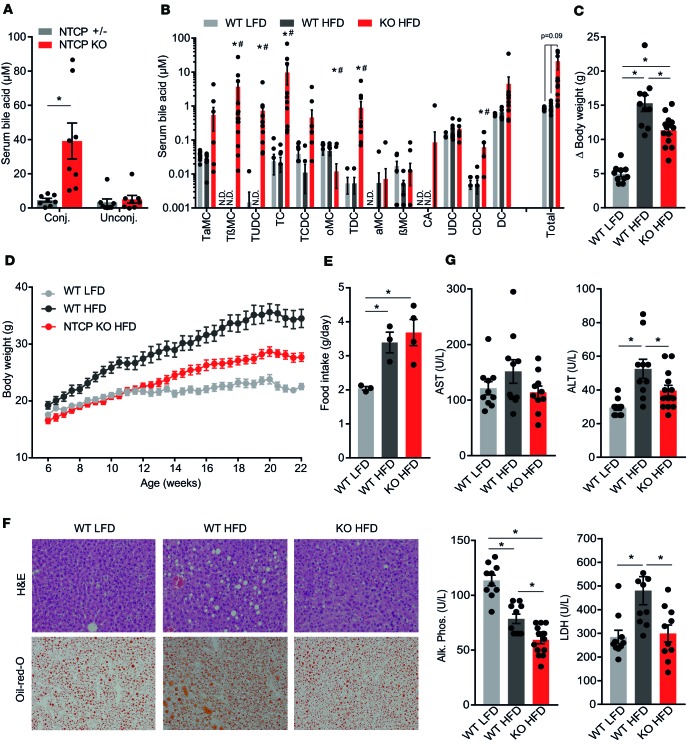
NTCP-KO mice have attenuated body weight gain and reduced (hepatic) adiposity when fed a high-fat diet. (**A**) Conjugated and unconjugated plasma bile acid levels, measured by high-performance liquid chromatography (HPLC), of nonfasted, chow-fed NTCP-expressing (^+/–^) and NTCP-KO mice (*n* = 8). conj., conjugated; unconj., unconjugated. (**B**–**G**) Female wild-type (WT, *n* = 10) or NTCP-KO (*n* = 13) mice were fed a low-fat diet (LFD) or high-fat diet (HFD) for 16 weeks. (**B**) Concentration of the individual conjugated and unconjugated bile acid species in plasma. Plasma was collected after a 4-hour fast, and bile acid concentration and species were measured by HPLC. N.D., not determined. Asterisk indicates significant changes of both HFD groups compared with the WT LFD group; hash tag indicates a significant change between NTCP-KO HFD and WT HFD mice. (**C**) Body weight change (Δ) and (**D**) body weight accumulation between the start and end of the experiment. (**E**) Food intake g/day per animal. Food intake per cage was weekly measured, divided over the number of animals per cage, and averaged for the 16-week period. *n* = 3 or 4 cages per group, each with 3 or 4 animals per cage. (**F**) Hepatic triglyceride content by representative images of liver histology by H&E (top) and Oil Red O (ORO, bottom) staining. Digital images were taken by using a ×10 eyepiece and a ×20 objective. (**G**) Plasma biochemistry displaying levels of aspartate aminotransferase (AST), alanine aminotransferase (ALT), alkaline phosphatase (Alk. Phos.), and lactate dehydrogenase (LDH). All data are represented as mean ± SEM; each dot represents an individual animal (**A**–**D** and **G**) or cage (**E**). *P < 0.05, calculated with 2-way ANOVA (Holm-Šídák’s) (**A**) or 1-way ANOVA (Tukey’s) (**B**–**E** and **G**).

Nonfasted NTCP-KO mice displayed increased conjugated plasma bile acid levels (Figure 1A) as a result of the delay in plasma clearance of conjugated bile acids (13). After a 4-hour fast conjugated plasma bile acid levels largely normalized to concentrations below 10 μM (Figure 1B), illustrating that the enhanced signaling was predominantly postprandial. To investigate the metabolic effect of prolonged postprandial bile acid elevations, we challenged NTCP-KO mice with a HFD. After 16 weeks, body weight gain was 26% lower in HFD-fed NTCP-KO mice compared with HFD-fed WT animals, irrespective of sex (Figure 1, C and D, females, and Supplemental Figure 1A, males; supplemental material available online with this article; https://doi.org/10.1172/jci.insight.127197DS1). WT animals displayed increased food intake upon HFD feeding compared with mice fed an LFD, but no difference in food intake was detected between WT and NTCP-KO groups fed a HFD (Figure 1E). In line with reduced body weight gain, HFD-fed NTCP-KO mice displayed decreased fat mass, predominantly in the subcutaneous white adipose tissue (sWAT) storage depot (Supplemental Figure 1B), and NTCP deficiency did not affect liver weight/body weight ratios (Supplemental Figure 1C). Liver steatosis was decreased in HFD-fed NTCP-KO mice compared with their HFD-fed WT counterparts (Figure 1F; Supplemental Figure 1, D–E, females; and Supplemental Figure 1F, males). Prolonged plasma bile acid signaling in NTCP-KO mice did not lead to liver damage as plasma levels of the liver enzymes aspartate aminotransferase (AST), alanine aminotransferase (ALT), alkaline phosphatase (ALP), and lactate dehydrogenase were normal, and HFD-induced elevations in liver enzyme values were completely prevented (Figure 1G).

**Figure 2:**
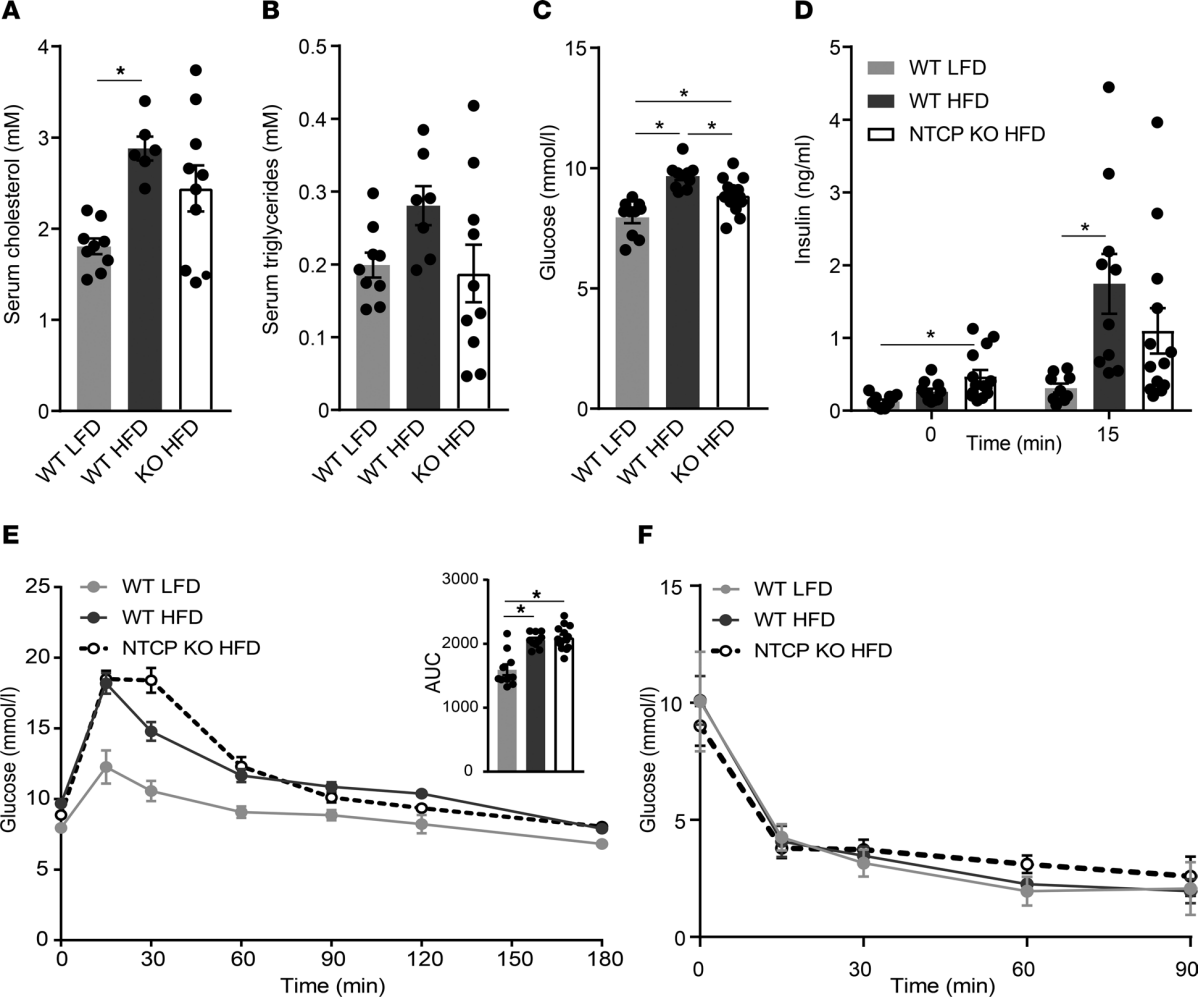
Improved key blood parameters but not glucose tolerance of NTCP-KO mice on HFD. Female WT (*n* = 10) or NTCP-KO (*n* = 13) mice were fed an LFD or HFD for 16 weeks. Animals were fasted for 4–5 hours before obtaining blood samples or performing glucose or insulin tolerance testing. (**A** and **B**) Plasma biochemistry displaying levels of triglycerides (**A**) and cholesterol (**B**). Plasma was analyzed by fast protein liquid chromatography (FPLC). (**C**–**F**) Fasting blood glucose (**C**) and plasma insulin levels (**D**) during an oral glucose tolerance test (OGTT, 2 g/kg glucose) (**E**) and an insulin tolerance test (1.2 mU/kg) (**F**). Glucose levels were determined in whole blood using a glucometer; plasma insulin was measured by ELISA. All data are represented as mean ± SEM; each dot represents an individual animal (**A**–**E**). *P < 0.05; 1-way ANOVA (Tukey’s) (**A**–**E**).

Because HFD feeding generally leads to a worsened plasma lipid and glucose profile, we assessed these key blood parameters in the NTCP-KO mouse model. The HFD-related increase in plasma cholesterol and the tendency toward increased plasma triglycerides in WT mice were both attenuated in the NTCP-KO animals (Figure 2, A and B). Furthermore, NTCP-KO mice fed a HFD had lower fasting glucose levels (Figure 2C). Next, we evaluated the effect of prolonged bile acid signaling on glucose tolerance. HFD-fed NTCP mice had moderate though significantly increased fasting insulin levels but reduced insulin levels 15 minutes after oral glucose administration (Figure 2D). This suggested improved insulin sensitivity for HFD-fed NTCP-KO mice, but this was not confirmed by an OGTT (Figure 2E) nor during an i.p. insulin tolerance test (Figure 2F).

### Reduced body weight of HFD-fed NTCP-KO mice is explained by decreased intestinal fat absorption and increased energy expenditure.

**Figure 3:**
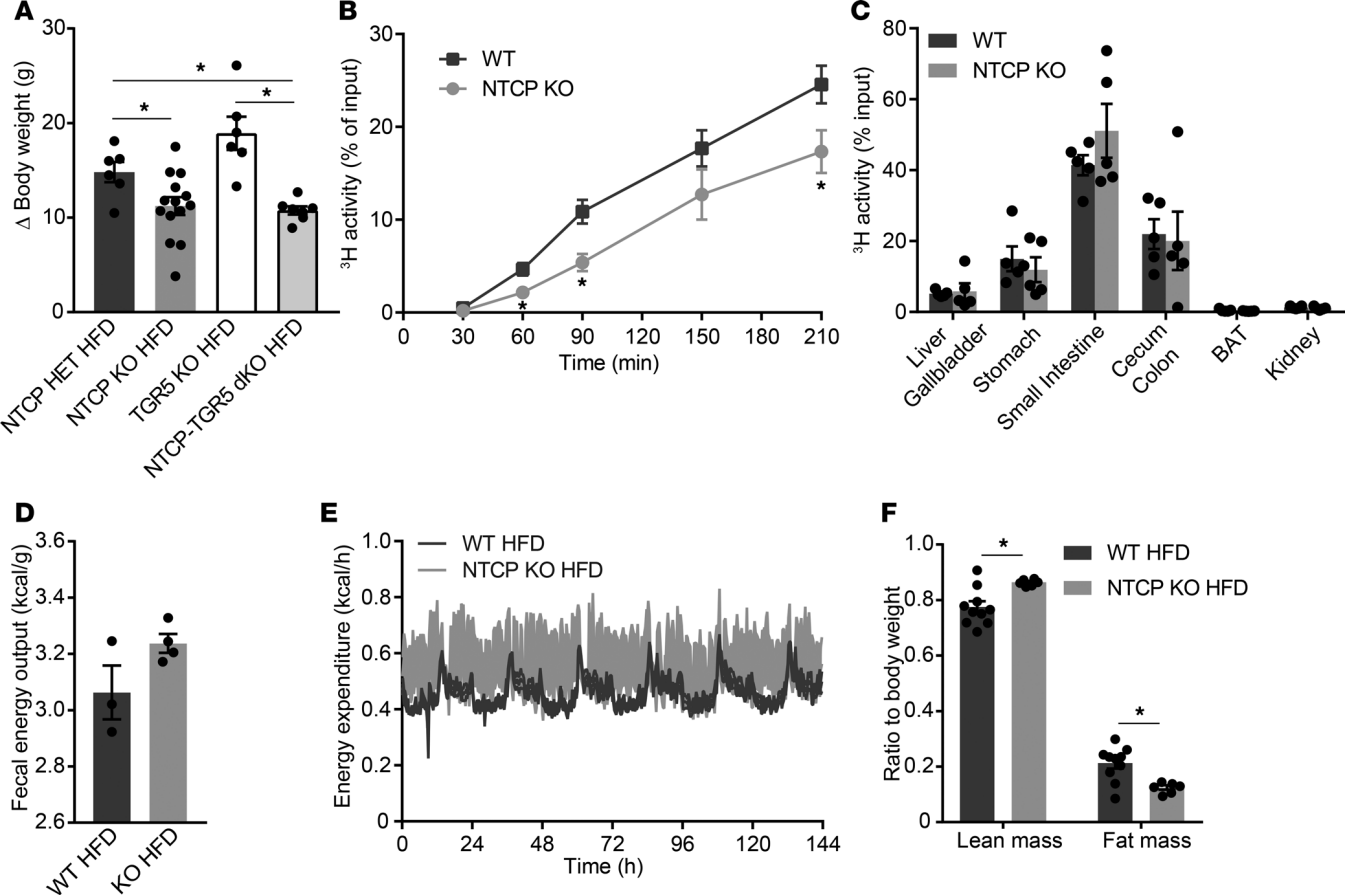
Enhanced bile acid signaling modulates intestinal fat absorption and increases energy expenditure. (**A**) Body weight change (Δ) of adult female control (NTCP-heterozygous), NTCP-KO, TGR5-KO, and NTCP-TGR5–double KO (NTCP-TGR5–dKO) mice after a 15-week HFD (*n* = 6–14 per group). (**B** and **C**) WT and NTCP-KO mice (*n* = 5 per group) were fasted 4 to 5 hours, after which they received an i.p. injection with Poloxamer 407 (1 mg/kg) to inhibit lipoprotein lipase. At *t* = 0, mice were orally gavaged with olive oil containing tracer. Amounts of [^3^H]triolein and ^3^H activity in whole blood (**B**) and organs (**C**) were determined by liquid scintillation counting. Blood volume was estimated as 4.706% of total body weight. BAT, brown adipose tissue. (**D**) Per cage, 24-hour feces were collected from HFD-fed WT and NTCP-KO mice (*n* = 3–4 cages per group, 3–4 animals per cage), and remaining fecal calories were assessed by bomb calorimetry. (**E** and **F**) WT (*n* = 10) and NTCP-KO (*n* = 6) mice fed a HFD for 3 weeks were individually housed in fully automated calorimetric cages. Energy expenditure was calculated from O_2_ consumption and the resting energy requirement (**E**), and lean and fat mass were assessed by NMR (**F**). Error bars show ± SEM; each dot represents an individual animal. *P < 0.05, calculated by 1-way ANOVA (**A**) or Student’s *t* test (**B** and **F**).

Because the reduction in body weight for HFD-fed NTCP-KO mice was not explained by differences in food intake (Figure 1E), we evaluated the potential mechanisms at play. First, we investigated the role of TGR5 because activation of this receptor leads to reduced body weight gain (17). Therefore, we generated mice that were deficient in both NTCP and TGR5 (NTCP-TGR5–double KO mice [NTCP-TGR5–dKO] mice). NTCP-TGR5–dKO mice were viable and fertile, and offspring showed a normal Mendelian frequency. After a 15-week HFD challenge, female NTCP-TGR5–dKO mice were equally protected against diet-induced obesity as mice deficient in NTCP only, while TGR5-KO mice gained even more weight than the controls (Figure 3A). Male NTCP-TGR5–dKO mice showed a similar but less pronounced trend toward reduced body weight gain compared to TGR5-KO animals (Supplemental Figure 2A). Plasma bile acid levels were similarly elevated in both NTCP-deficient groups, and the TGR5-KO–associated increase in (hepatic) adiposity was prevented in NTCP-TGR5–dKO mice (Supplemental Figure 2, B–D). Next, we determined the metabolic effect of NTCP inhibition on intestinal fat absorption and energy expenditure. NTCP-KO mice had decreased uptake of orally administered fatty acids from the small intestine (Figure 3B), with a trend toward more triolein remaining in the small intestine compared with their WT controls (Figure 3C and Supplemental Figure 2E). Bomb calorimetric assays of feces demonstrated a tendency toward decreased caloric extraction from the diet by NTCP-KO mice placed on a HFD for 16 weeks (Figure 3D), but caloric extraction was not affected in a short-term HFD feeding regimen when mice were individually housed (Supplemental Figure 2F). Next, we assessed the contribution of energy expenditure to the attenuated weight gain in HFD-fed NTCP-KO mice by means of indirect calorimetry. A profound increase in energy expenditure was observed in HFD-fed NTCP-KO mice compared with their HFD-fed WT counterparts (Figure 3E). The increase in energy expenditure was not explained by changes in ambulatory activity (Supplemental Figure 3A) and food intake was unchanged. The respiratory quotient remained similar for NTCP-KO mice (Supplemental Figure 3B), pointing toward a similarly increased oxidation rate of both carbohydrates and fatty acids. Effects on body weight, adiposity, and serum bile acid levels were in line with the results of long-term HFD feeding (Supplemental Figure 3, C–E). Additionally, we specifically assessed body composition by nuclear magnetic resonance (NMR), showing that HFD-NTCP-KO mice had a lower fat mass and higher lean mass relative to their total body weight (Figure 3F). Of note, correction for lean body mass had no effect on the results for energy expenditure (Supplemental Figure 3F). The phenotype of increased energy expenditure was also observed in young chow-fed NTCP-KO mice, again accompanied by a decreased fat mass and increased lean mass compared with body weight (Supplemental Figure 4, A–C). Similarly to the metabolic assessment of HFD-NTCP-KO mice, increased energy expenditure was not explained by differences in ambulatory activity, nor was the respiratory quotient changed (Supplemental Figure 4, D and E).

### Increased energy expenditure is a result of brown adipose tissue thermogenesis.

**Figure 4:**
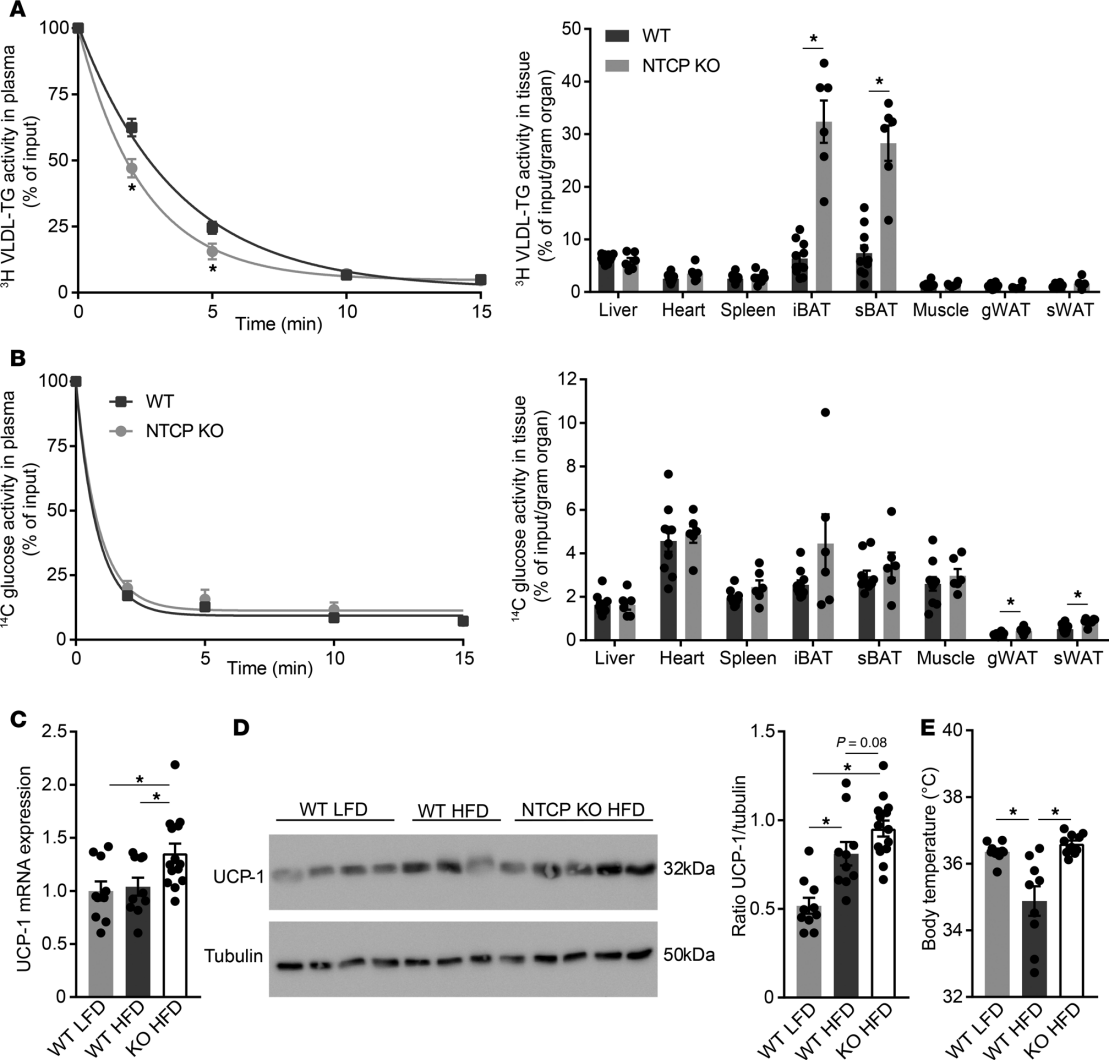
Prolonged bile acid signaling stimulates BAT uncoupled respiration. (**A** and **B**) 3-week HFD-fed WT (*n* = 10) and NTCP-KO mice (*n* = 6) were fasted 4 to 5 hours and subsequently i.v. injected with radiolabeled [^14^C]deoxyglucose and [^3^H]triolein-labeled VLDL-like particles. Plasma clearance and uptake by organs at 15 minutes after injection were determined by assessing ^3^H and ^14^C activity by liquid scintillation counting. Blood volume was estimated as 4.706% of total body weight. iBAT, interscapular brown adipose tissue; sBAT, supraclavicular brown adipose tissue; gWAT, gonadal white adipose tissue. (**C** and **D**) Uncoupling protein 1 (*Ucp1*) mRNA (**C**) and UCP1 protein (**D**) expression levels, determined by reverse transcription quantitative PCR (RT-qPCR) and Western blotting, respectively, in BAT of WT and NTCP-KO mice fed an LFD or HFD for 16 weeks (*n* = 10–13). RT-qPCR samples are relative to the geometric mean of control genes *36b4* and *Hprt* and were normalized to WT LFD. The Western blot shown in **D** is representative of all mice, and for each mouse the relative UCP1 (32 kDa) to tubulin (50 kDa) protein expression was determined. (**E**) Body temperature, measured by temperature transponders, of the mice in **C** and **D**. Per animal, average body temperature was calculated from 10 individual observations. Error bars show ± SEM; each dot represents an individual animal. *P < 0.05, calculated by Student’s *t* test (**A** and **B**) or 1-way ANOVA (**C**–**E**).

**Figure 5:**
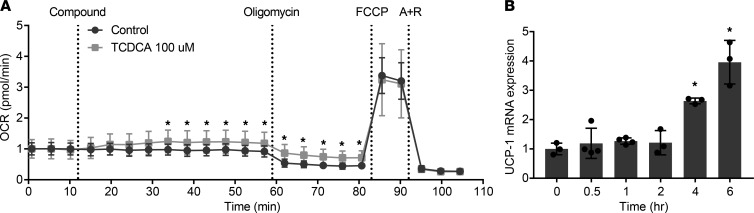
Prolonged bile acid signaling increases uncoupled respiration in SGBS cells. (**A**) Oxygen consumption rate (OCR) in SGBS cells measured by the XFe 96 extracellular flux analyzer. Cells were stimulated with the bile acid taurochenodeoxycholic acid (TCDCA) or vehicle (control) after the first 3 measurements. Subsequently, activators and inhibitors of the mitochondrial respiratory chain were applied at the indicated time points. FCCP, carbonyl cyanide-4-(trifluoromethoxy)phenylhydrazone; A + R, antimycin A + rotenone. Data are represented as OCR normalized to basal respiration (average of 3 cycles). *n* = 14, representative results of triplicate experiments. (**B**) *UCP1* mRNA expression level in SGBS cells treated with bile acid TCDCA. Samples are relative to the geometric mean of β-actin and *HPRT* and subsequently normalized to the expression at time point 0 (representative results of triplicate experiments, *n* = 3–4 wells/group). Error bars show mean ± SD; each dot represents an individual sample. Student’s *t* test (**A**) or 1-way ANOVA using Dunnett’s multiple-comparisons test (**B**) was used to calculate significance; *P < 0.05.

To identify the organ(s) involved in the increased energy expenditure and redistribution of fat/lean mass, we determined the tissue distribution after i.v. injection of a mixture of radiolabeled triglyceride-derived fatty acids packaged in VLDL-like particles and radiolabeled deoxyglucose. In HFD-fed NTCP-KO mice, plasma clearance of triglycerides was faster than in HFD-fed WT animals, with *t_1/2_* = 1.71 ± 0.16 minutes for NTCP-KO versus 2.64 ± 0.23 minutes for WT (Figure 4A and Supplemental Figure 5A). Plasma clearance of deoxyglucose was unchanged between the groups (Figure 4B and Supplemental Figure 5B). Adipose tissues of HFD-fed NTCP-KO mice proved more metabolically active as evidenced by increased glucose uptake in both subcutaneous and gonadal white adipose tissue and a 9.7 ± 1.2–fold increased uptake of triglyceride-derived fatty acids per gram of BAT when compared with HFD-fed WT animals (Figure 4, A and B). In the NTCP-KO group, total capacity to take up the radiolabeled triglycerides from plasma was increased for BAT and heart, and deoxyglucose uptake was increased in subcutaneous adipose tissue, together with the spleen, together underscoring the increased activity of adipose tissues in HFD-fed NTCP-KO mice (Supplemental Figure 5, C and D). BAT is specialized in burning fat as a fuel through uncoupling mitochondrial oxidation (18) and known to be stimulated by bile acid supplementation in rodents (4, 19) and humans (20). Gene expression analysis of BAT indicated an increased expression of mitochondrial genes associated with energy expenditure and thermogenesis, such as uncoupling protein 1 (*Ucp1*) (Figure 4C and more genes in Supplemental Figure 5E), and UCP1 levels showed an increased trend in NTCP-KO mice (Figure 4D). Concordantly, we observed an increased body temperature (Figure 4E). Subsequently, the role of bile acids in energy expenditure was investigated in a human brown fat cell line using the Simpson-Golabi-Behmel syndrome (SGBS) preadipocyte cell line, which has high UCP1 expression levels when fully differentiated (21, 22). Addition of the conjugated bile acid taurochenodeoxycholic acid (TCDCA) rapidly increased the oxygen consumption rate, with 20%–25% in the basal respiration phase, (reflecting an ~35% increase in mitochondria-dependent respiration), and this elevation remained after the addition of oligomycin (Figure 5A), a compound that blocks ATP synthesis–linked respiration. TCDCA did not induce further changes in respiration rate after addition of FCCP (maximal respiration) and rotenone with antimycin A (leaving only nonmitochondrial respiration intact). Furthermore, within hours TCDCA induced increased mRNA expression of *UCP1* (Figure 5B). Together, this suggests that the systemic overflow of bile acids caused by NTCP inhibition increases energy expenditure by BAT thermogenesis, likely resulting from an increase in mitochondrial uncoupling.

## Discussion

In this study, we demonstrate that deletion of the hepatic bile acid transporter NTCP prolongs the postprandial elevation in plasma bile acids and ameliorates diet-induced obesity. In the past, it has been shown that pharmacologic bile acid receptor activation results in metabolic improvements (4–7), but the specific and continuous action of receptor agonists is not in line with the natural situation of bile acid receptor activation in the short time frame after a meal, at which plasma bile acid levels are temporarily increased (11). Here, we developed a strategy to create a prolonged postprandial peak in plasma bile acid levels by deletion of NTCP and in this way stimulated bile acid signaling with the endogenous ligands in a natural rhythm. Notably, early metabolic improvements observed in bariatric surgery patients are also accompanied by increased bile acid levels (23) and in mice and pigs are associated with reduced NTCP expression (24, 25).

Glucose excursions upon glucose and insulin injections were similar between WT and NTCP-KO mice, despite the reduced fasting glucose levels in the latter. This discrepancy might be due to altered intracellular bile salt signaling in the liver and subject to future investigations. Both NTCP-KO and NTCP-TGR5–dKO mice displayed attenuated weight gain upon high-fat feeding. Although TGR5 has been established as an important receptor influencing bile acid–induced energy expenditure (4, 17), it turned out not to be essential for the bile acid–mediated metabolic effects in our model. Meanwhile, increased energy expenditure, possibly complemented by decreased intestinal fat absorption, can explain the lower body weight of NTCP-KO mice. How (conjugated) bile salts signal to increase energy expenditure in the absence of TGR5 remains unclear and might involve another bile salt receptor. Increased BAT activation results in increased nonshivering thermogenesis via UCP1 (26, 27), in line with what we found here in NTCP-KO mice. BAT activation promotes fat oxidation and enhances both glucose and triglyceride uptake from the bloodstream (27–29), which was reflected by increased triglyceride clearance from the systemic circulation and extensive triglyceride-derived fatty acid uptake in BAT in NTCP-KO mice. The increased uptake of glucose into sWAT and gonadal white adipose tissue (gWAT) possibly points toward increased oxidative capacity in these 2 tissues as well, especially because browning of beige adipocytes in white adipose tissue has recently been presented as an interesting contributor to nonshivering thermogenesis (30–32). Because BAT activity controls triglyceride clearance (28), it is tempting to speculate that its need for substrates drives mobilization of lipids from other tissues, which then can explain the decrease in hepatic but also sWAT triglyceride content in the current study. The recent discovery that not only rodents and human neonates but also human adults have metabolically active BAT (33, 34) presents BAT thermogenesis as an attractive approach to enhance energy expenditure and complement existing strategies to induce weight loss. Bile acid–mediated stimulation of BAT has been shown in humans (20), underscoring the relevance of the results obtained in the study presented here.

In conclusion, prolonged postprandial elevation of plasma bile acid levels by decreasing hepatic bile acid uptake is a potentially novel means to attenuate body weight gain, and reduce liver and body fat mass, by increased BAT thermogenesis complemented by reduced intestinal calorie uptake.

## Methods

### Animals and experimental design.

NTCP-deficient (*Slc10a1*-KO, C57BL/6 background; ref. 13), TGR5-KO (*Gpbar1*-KO, C57BL/6 background; ref. 17), and NTCP-TGR5–dKO mice (*Slc10a1-Gpbar1*-dKO, C57BL/6 background) were bred in the Academic Medical Center, Amsterdam, the Netherlands. Control WT C57BL/6JOlaHsd mice were purchased from Envigo. NTCP-heterozygous littermates were used as controls in the NTCP-TGR5–dKO HFD experiment. Experiments were performed at the Academic Medical Center, except for the calorimetric study with NTCP-KO animals, which was performed in the Leiden University Medical Center. Animals were cohoused with 3–4 animals and kept on a 12-hour light/12-hour dark continuous cycle (700–1900 hours) with ad libitum access to food and water. For the calorimetric studies, animals were individually housed in PhenoMaster cages (TSE Systems). After 2 days of acclimatization, O_2_ consumption, CO_2_ production, and caloric intake were measured for 4 consecutive days. Total energy expenditure was estimated from the VO_2_ and resting energy requirement. Respiratory quotient was calculated as amount of CO_2_ produced divided by the amount of O_2_ consumed. Physical activity was monitored using infrared sensor frames. During the calorimetric studies, total body fat and lean mass were monitored with NMR using the EchoMRI-100 (EchoMRI). Animals were fed a standard rodent chow (Envigo), HFD (D12492, rodent diet with 60 kcal% fat; Research Diets, Inc.), or matching LFD (D12450B, rodent diet with 10 kcal% fat; Research Diets, Inc.). Female mice were 3–4 or 7–8 weeks old at the start of the experiment, and male mice were 8–12 weeks old, except for the calorimetric measurements in young NTCP-KO and WT animals, which had an age of 3–6 weeks at the start of the experiment. Body weight was monitored twice weekly and food intake once weekly. Animal temperature data were obtained by noninvasive readout of IPTT-300 temperature transponders (BMDS) subcutaneously implanted in the flank of each animal. Glucose and insulin tolerance testing were performed 1 week before sacrifice. At the end of the study, a fasting (4–5 hours) blood sample was taken, and organs were harvested to be frozen in liquid nitrogen or formalin fixed.

### Chemicals.

Bile acid TCDCA and lipase inhibitor Poloxamer 407 were purchased from MilliporeSigma. [^3^H]Triolein (0.5 mCi/mL) and [^14^C]deoxyglucose (0.01 mCi/mL) were purchased from Perkin Elmer. We obtained d-glucose from Merck and human insulin (Humulin R U-100) from Lilly.

### Glucose and insulin tolerance testing.

Animals were fasted 4–5 hours before the experiment. Mice received glucose (2 g/kg) by oral gavage or insulin (1.2 mU/kg) by i.p. injection. Blood was drawn by the tail vein at 0, 15, 30, 60, and 90 minutes and for the OGTT also at 120 minutes. Glucose levels were determined in whole blood using a Contour XT glucometer (Bayer B.V.). Insulin levels were measured in plasma using the Ultra Sensitive Mouse Insulin ELISA kit (Chrystal Chem).

### Intestinal lipid absorption.

This experiment was conducted in 6- to 12-week-old male NTCP-KO mice and their respective C57BL/6JOlaHsd controls of the same age and sex. Animals were fasted 4–5 hours, after which they received an i.p. injection with Poloxamer 407 (1 mg/kg) to inhibit triglyceride hydrolysis by lipoprotein lipase. Mice were orally gavaged with olive oil containing tritium-labeled triolein (1 μCi/animal). The next 4 hours, for every 30 minutes 5 μL whole blood was sampled via the tail vein and collected in microscintillation vials containing 100 μL 0.1 M EDTA, 50 μL 3% H_2_O_2_, and 50 μL Solvable (Perkin Elmer). The experiment was terminated by heart puncture; organs were collected, weighted, and overnight dissolved in 1 mL Solvable (Perkin Elmer) at 37°C. Organ samples were decolored the next day by addition of 200 μL 30% H_2_O_2_, incubated for 1 hour at 50°C, and transferred to microscintillation vials. Radioactivity in organs and blood was measured by liquid scintillation counting using the TRI-CARB 2900 TR (Perkin Elmer). Plasma volume was estimated at 4.706% of total body weight (35–40).

### VLDL and glucose clearance experiment.

NTCP-KO and C57BL/6JOlaHsd controls were fasted for 4 hours and injected via the tail vein with radiolabeled VLDL-like particles and glucose (2 μCi [^3^H]triolein and 0.5 μCi [^14^C]deoxyglucose per animal). Preparation of 80-nm-sized VLDL-like particles was done as described previously (41). Sampling and analysis were performed as described previously (40). Plasma volume was estimated at 4.706% of total body weight (35–40).

### Fecal energy.

Energy excreted in feces was measured using a bomb calorimeter (IKA C1). Feces were compacted and dried in a freeze dryer (LabConcol, Beun de Ronde) overnight before combustion.

### Cell culture.

SGBS human preadipocyte cells provided by Eric Kalkhoven (Center for Molecular Medicine and Department of Molecular Cancer Research, University Medical Center Utrecht, Utrecht University, Utrecht, the Netherlands) and Martin Wabitsch (Division of Pediatric Endocrinology and Diabetes, University Medical Center Ulm, Ulm, Germany) were grown in DMEM/F12 (Lonza) supplemented with 10% fetal calf serum (Gibco) and 1% penicillin/streptomycin (pen/strep, Lonza). Cells were passaged twice a week at a confluence of 70%–80%, with a maximum of 10–12 passages, and incubated in a humidified atmosphere of 37°C and 5% CO_2_. SGBS cell differentiation was performed in 24-well (734-2325, VWR) or Seahorse 96-well (XF96, Seahorse Bioscience) plates by overconfluent seeding (55,000 or 2000 cells/well in a 24-well or Seahorse 96-well plate, respectively). After 3 days, medium was refreshed with differentiation mix 1, consisting of DMEM/F12 supplemented with 1% pen/strep, 8 mg/mL biotin (FB01, Thermo Fisher Scientific), 4 mg/mL pantothenate (FP10, Santa Cruz Biotechnology), 0.12 mg/mL insulin (MilliporeSigma), 10 mg/L transferrin (Roche), 1 μM triiodothyronine (T3, MilliporeSigma), 2 μM rosiglitazone (MilliporeSigma), 500 μM 3-isobutyl-1-methylxanthine (IBMX, MilliporeSigma), and 1 μM dexamethasone (MilliporeSigma). Medium was refreshed 96 hours later with differentiation mix 2, similar to mix 1 but without T3, rosiglitazone, IBMX, and dexamethasone. Experiments were performed another 96 hours later.

### SGBS mitochondrial respiration.

SGBS cells were differentiated in Seahorse 96-well plates as described above. The plate was incubated in prepared assay medium (2× diluted 2xBase Medium supplemented with 25 mM glucose, 1 mM Na pyruvate, and 2 mM l-glutamine, pH 7.4, set at 37°C with 2 M NaOH) for 1 hour in a non-CO_2_ incubator at 37°C before measuring in an XFe 96 extracellular flux analyzer (Seahorse Bioscience). The sensor cartridge was hydrated in XF Calibrant at 37°C in a water bath overnight. OCR was measured over 4-minute periods with a mixing of 2 minutes in each cycle, with 23 cycles in total. Inhibitors and activators were used at the following concentrations: oligomycin (1.5 μM), FCCP (1 μM), antimycin A (2.5 μM), rotenone (1.25 μM), and TCDCA (100 μM). Data are represented as OCR normalized to basal respiration (average of 3 cycles). All chemicals and materials for the XF Cell Mito Stress Test were obtained from Agilent Seahorse Bioscience, unless stated otherwise.

### Bile acid cell signaling study.

Differentiated SGBS cells in 24-well plates were incubated with differentiation mix 2 supplemented with 10 μM bile acid TCDCA. After 0, 60, 120, 240, or 360 minutes, medium was removed, and cells were harvested in 500 μL TRI Reagent (MilliporeSigma) for RNA isolation.

### RNA isolation and RT-qPCR.

Total RNA was isolated from cells (1 well of a 24-well plate) or approximately 50 mg of tissue with TRI Reagent (MilliporeSigma). RNA integrity was assessed spectrophotometrically at 260 nm using a NanoDrop 1000 (Thermo Fisher Scientific). One thousand nanograms of total RNA was treated with DNAse (Promega), and first-strand cDNA was synthesized with oligo-dT and Revertaid reverse transcriptase (Fermentas). RT-qPCR was carried out in a Roche LightCycler 480 II instrument using SensiFAST SYBR No-ROX kit (Bioline) and was analyzed using LinRegPCR 12.5 software (42). Expression levels in each sample were normalized for the geometrical mean of 2 reference genes. Primer sequences are noted in Supplemental Table 1.

### Histopathology.

Formalin-fixed, paraffin-embedded liver or adipose tissue samples, 4.5-μm-thick sections, were stained with hematoxylin (MilliporeSigma, 51275) and eosin (MilliporeSigma, E4382) (H&E). From the snap-frozen liver tissue, 5-μM-thick cryosections were fixed with 3.7% formaldehyde (MilliporeSigma) for 60 minutes and stained with ORO (MilliporeSigma) for 30 minutes. Digital imaging of all sections was performed using an Olympus BX-51 microscope equipped with a ×10 eyepiece and a ×20 objective. The amount of liver fat stained by ORO was calculated from 4 random pictures taken from each cryosection using ImageJ software (NIH).

### Liver triglycerides.

Hepatic lipids were extracted by a chloroform/methanol extraction protocol as described previously (43). Liver triglycerides were measured using the Trig/GB-kit (Roche).

### Plasma biochemistry.

Plasma biomarkers for liver injury (ALT, AST) and cholestatic parameters (ALP) were determined by routine clinical biochemistry testing on a Roche Cobas c502/702 analyzer (Roche Diagnostics).

Total cholesterol and triglyceride content in the main lipoprotein classes (VLDL, LDL, and HDL) was determined using FPLC. The system contained a PU-980 ternary pump with an LG-980-02 linear degasser, FP-920 fluorescence, and UV-975 UV/VIS detectors (Jasco). An extra PU-2080i Plus pump (Jasco) was used for in-line cholesterol PAP or triglyceride enzymatic reagent (Roche) addition at a flow rate of 0.1 mL/min. Plasma lipoproteins were separated using a Superose 6 Increase 10/30 column (GE Healthcare) using TBS, pH 7.4, as the eluent at a flow rate of 0.31 mL/min. Commercially available lipid plasma standards (low, medium, and high) were used for generation of total cholesterol or triglycerides calibration curves for the quantitative analysis (Stichting Kwaliteitsbewaking Medisch Laboratoriumonderzoek [SKML]) of the separated lipoprotein fractions. Quantitative analysis of the chromatograms was carried out with Chrom Nav chromatographic software, version 1.0 (Jasco).

### Western blot.

Frozen BAT was pestle homogenized on ice in RIPA buffer (50 mM Tris-HCl at pH 8.0, 150 mM NaCl, 1% *v/v* NP-40, 0.5% *w/v* Na deoxycholate, 0.1% *w/v* SDS) supplemented with protease inhibitor cocktail (Roche). Samples were separated on a 10% SDS-PAGE (50 μg protein/lane) and transferred by semidry blotting to a PVDF membrane. Proteins were probed with rabbit anti-UCP1 (1:1000; ab10983, Abcam) and rabbit anti–β-tubulin (1:1000; 2148, Cell Signaling). Immune complexes were detected with a horseradish peroxidase–conjugated secondary antibody (Bio-Rad), visualized using enhanced chemiluminescence detection reagent (Lumi-Light, Roche), and detected using ImageQuant LAS 4000 (GE Healthcare).

### Statistics.

Data are provided as the mean ± SEM (in vivo experiments) or SD of the mean (in vitro experiments). Differences between 2 groups were analyzed using 2-tailed Student’s *t* test; 1-way ANOVA with Tukey’s or Dunnett’s post hoc analysis was used for comparisons of multiple groups. Statistical significance was set at *P* < 0.05, and calculations and graphs were generated using GraphPad Prism 7.0 (GraphPad Software Inc.).

### Study approval.

The study design and animal care and handling were approved by the Institutional Animal Care and Use Committee of the University of Amsterdam and of the Leiden University Medical Center.

## Author contributions

JMD carried out experiments. SK performed the calorimetric and clearance experiments in Leiden, under the supervision of PCNR. DS was involved in the intestinal lipid absorption experiments, RFK performed bomb calorimetry on fecal samples, and LH performed liver histology. HPLC and FPLC for plasma bile acid or plasma triglycerides and cholesterol analysis were operated by DRDW and JHML, respectively. JMD, RPJOE, and SFJVDG developed the study concept and design. Drafting and initial review of the manuscript were performed by JMD, SK, DS, RFK, RLPRA, DRDW, JHML, KS, PCNR, RPJOE, and SFJVDG. SFJVDG and PCNR obtained funding. All authors were involved in analysis and interpretation of data and have read and approved the manuscript.

## Supplementary Material

Supplemental data

## Version 1 06/25/2019

In-Press Preview

## Version 2 07/25/2019

Electronic publication
