# Supplementary material for: NTCP deficiency in mice protects against obesity and hepatosteatosis
Source: JCI Insight. 2019 Jul 25;4(14):e127197. doi: 10.1172/jci.insight.127197 (PMC6675549; doi:10.1172/jci.insight.127197)
Supplement: Supplemental data [file jciinsight-4-127197-s059.pdf]

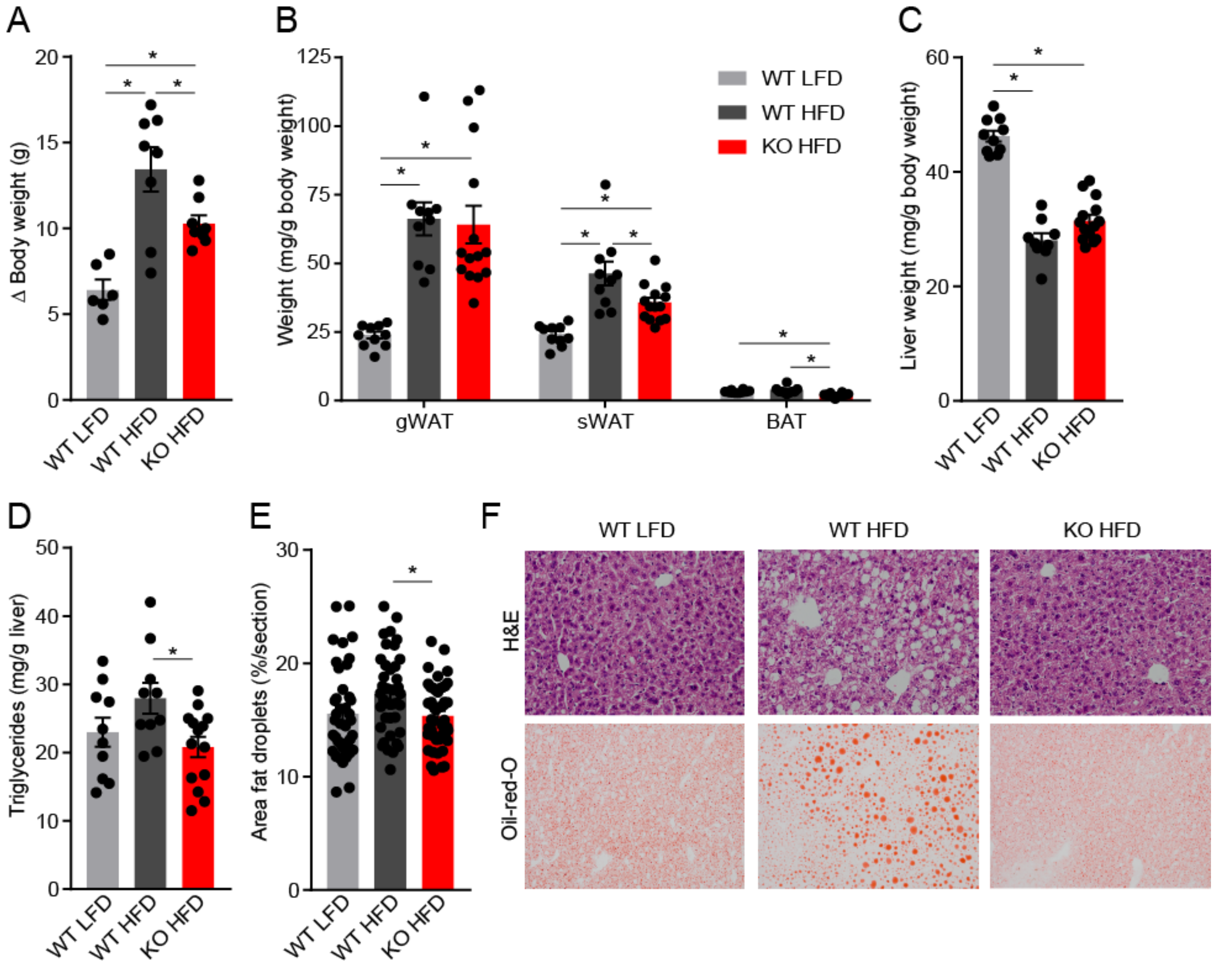

Figure S1. Insights into male body weight, fat distribution, and liver triglycerides of NTCP KO mice on HFD. (A) Body weight change (Δ delta) of male WT or Na^+-taurocholate co-transporting polypeptide (NTCP) KO mice fed a low fat diet (LFD) or high fat diet (HFD) for 16 weeks (n=6-8 mice/group). (B-E) Female WT (*n* = 10) or NTCP KO (*n* = 13) were fed a LFD or HFD for 16 weeks. Graphs display weights, expressed as ratio to total body weight, of fat compartments (B), gonadal and subcutaneous white adipose tissue (gWAT, sWAT) and brown adipose tissue (BAT), and theliver (C). (D-E) Liver triglyceride content determined by biochemical chloroform/methanol extraction (D) and oil-red-o staining (E) (n=4 images/mouse). (F) Hepatic triglyceride content by representative images of liver histology by H&E (top) and oil-red-o (bottom) staining of the mice described in (A). Digital images were taken by using a x10 eyepiece and a x20 objective. All data are represented as mean ± SEM, each dot represents an individual animal. *P<0.05, calculated by 1-way ANOVA (Tukey's multiple comparison).

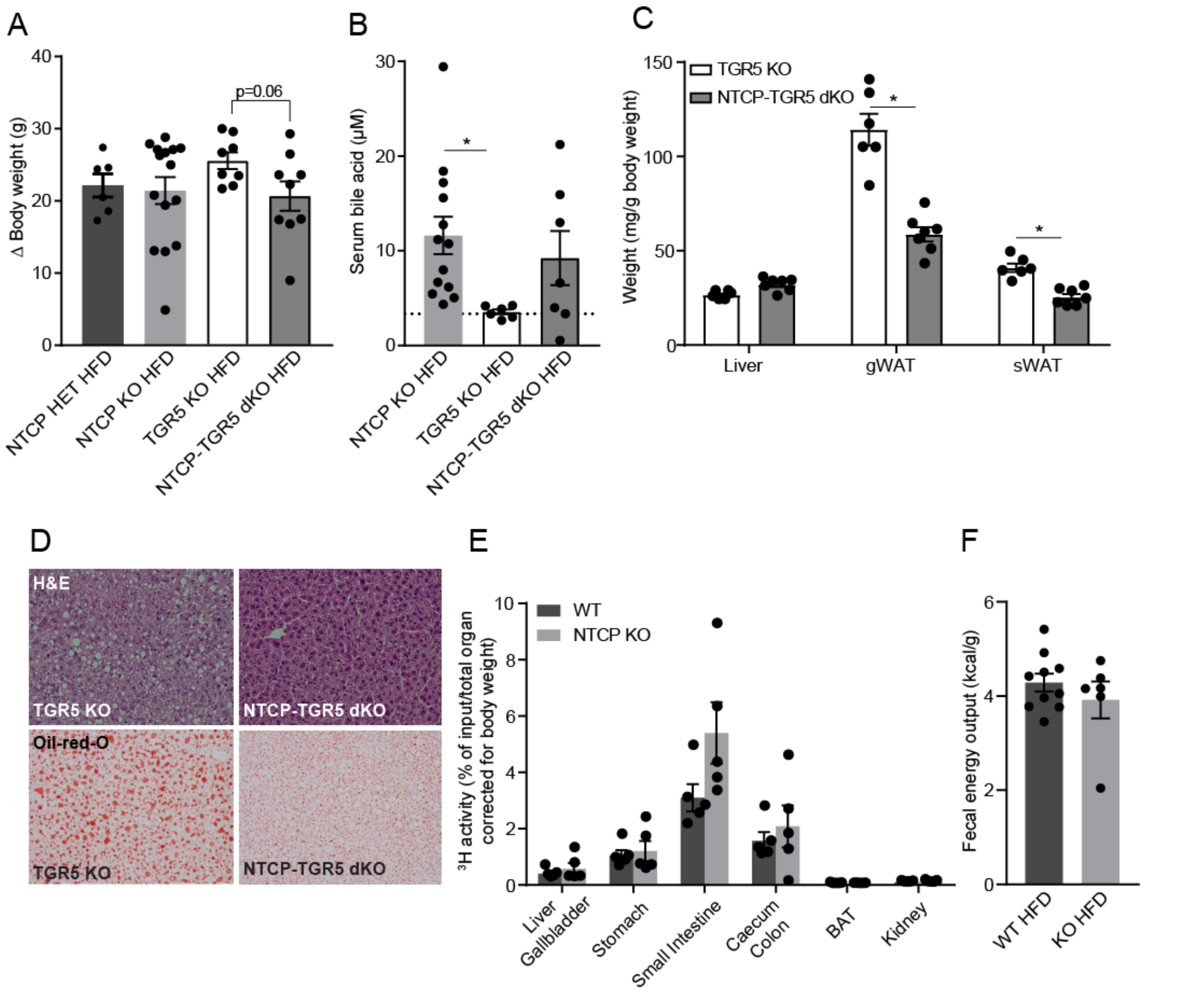

Figure S2. TGR5 is not essential in the protection against diet-induced-obesity of NTCP deficient mice. (A) Body weight change (Δ delta) of adult male control (Na+-taurocholate co-transporting polypeptide (NTCP) heterozygous), NTCP KO, G protein-coupled bile acid receptor (TGR5) KO, and NTCP-TGR5 double KO (dKO) mice after a 15-week high fat diet (HFD) (*n* = 6-15 per group). (B-D) Adult female control (NTCP heterozygous), NTCP KO, TGR5 KO, and NTCP-TGR5 dKO mice were fed a HFD for 15 weeks, (*n* = 6-14 per group). (B) Total plasma bile acid levels after a 4 hour fast were measured by high-performance liquid chromatography (HPLC). (C) Graphs display weights, expressed as ratio to total body weight, of the fat compartments, gonadal and subcutaneous white adipose tissue (gWAT, sWAT) and, brown adipose tissue (BAT). (D) hepatic triglyceride content by representative images of liver histology by H&E (top) and oil-red-o (bottom) staining. (E) WT and NTCP KO mice (*n* = 5 per group) were fasted 4-5 hours after which they received an i.p. injection with Poloxamer 407 (1 mg/kg) to inhibit lipoprotein lipase. At t=0, mice were orally gavaged with olive oil containing tracer amounts of [^{3}H]triolein and organs were harvested 3.5 hours later. ^{3}H activity in organs was determined by liquid scintillation counting and data was corrected for organ and total body weight. (F) 3-week HFD-fed WT and NTCP KO mice (*n* = 6-10) were individually housed and feces was collected for 24 hours. Remaining fecal calories were assessed by bomb-calorimetry. All data are represented as mean ± SEM, each dot represents an individual animal. *P<0.05, calculated by 1-way ANOVA (Tukey's multiple comparison).

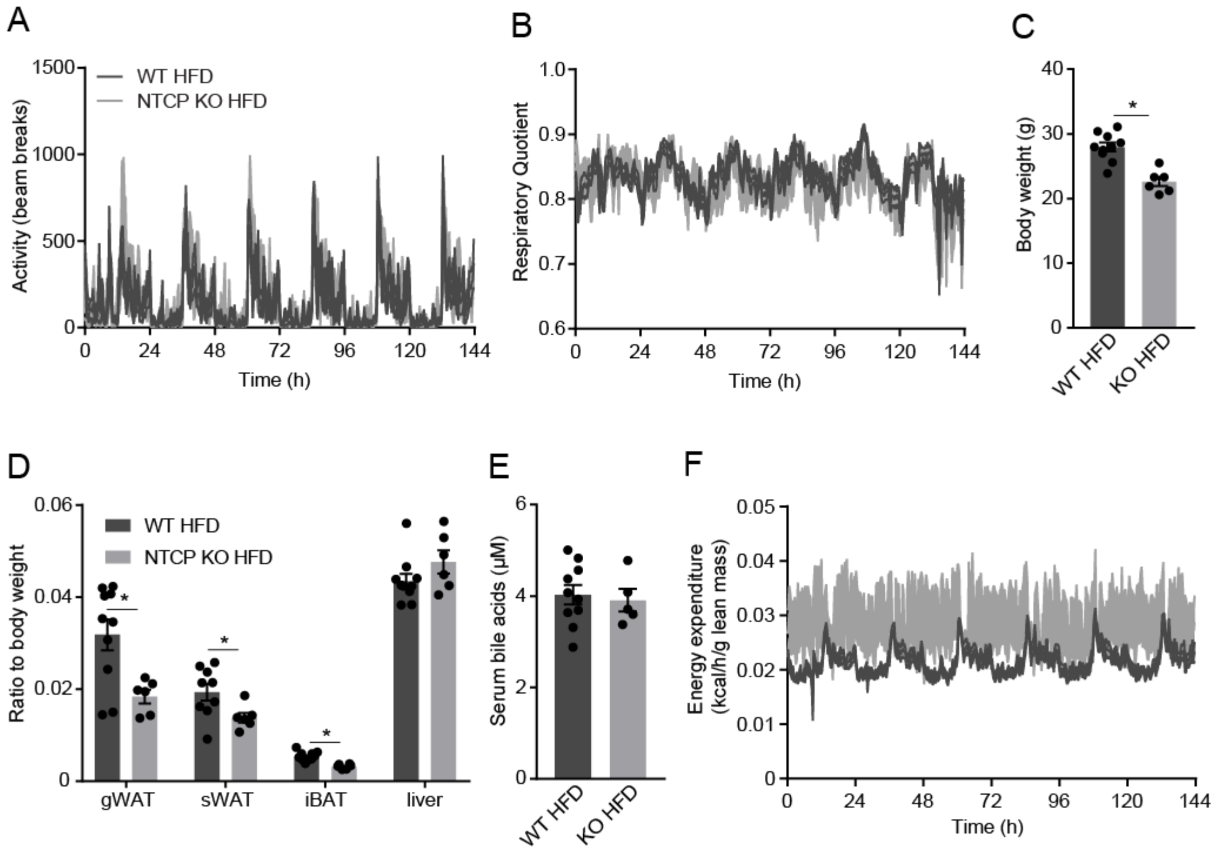

Figure S3. Enhanced bile acid signaling in HFD-fed NTCP KO increases energy expenditure. (A-F) WT (*n* = 10) and Na+-taurocholate co-transporting polypeptide (NTCP) KO (*n* = 6) mice fed a HFD for 3 weeks were individually housed in fully automated calorimetric cages. Locomotor activity was monitored using infrared sensor frames (A) and respiratory quotient was calculated as amount of CO_2 produced divided by the amount of O_2 consumed (B). Body weight (C) and organ weight expressed as ratio to total body weight, of the fat compartments, gonadal and subcutaneous white adipose tissue (gWAT, sWAT) and, brown adipose tissue (BAT) (D). (E) Total plasma bile acid levels after a 4 hour fast were measured by high-performance liquid chromatography (HPLC). (F) Energy expenditure was calculated from O_2 consumption and the resting energy requirement and corrected for lean mass. All data are represented as mean ± SEM, each dot represents an individual animal. *P<0.05, calculated by student t-test.

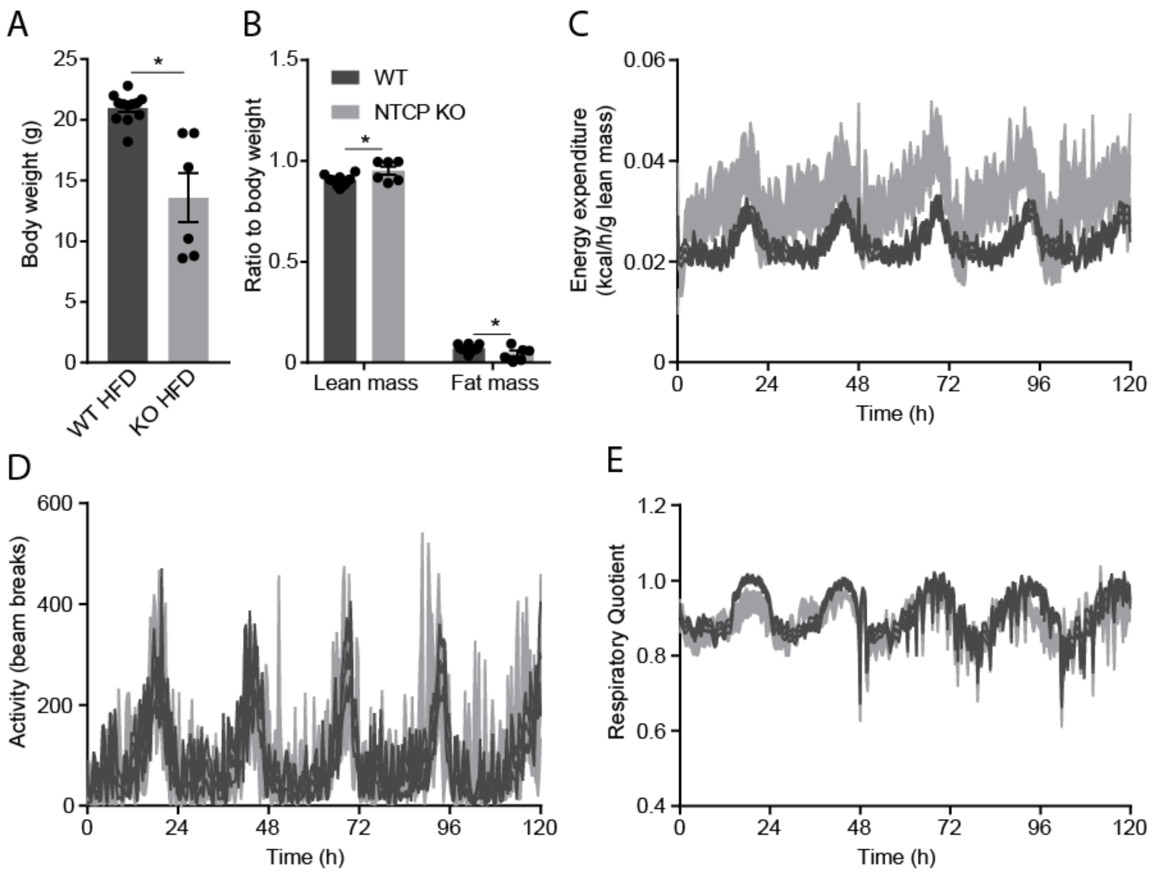

Figure S4. Energy expenditure is increased in young chow-fed NTCP KO mice. (A-E) Chow-fed WT and Na+-taurocholate co-transporting polypeptide (NTCP) KO mice of 4 weeks of age were individually housed in fully automatic calorimetric cages (*n* = 6-10 group). Total body weight (A) and lean and fat mass assessed by NMR (B). Energy expenditure was calculated from the O_2 consumption and the resting energy requirement and corrected for lean body weight (C). Locomotor activity was monitored using infrared sensor frames (D) and respiratory quotient was calculated as amount of CO_2 produced divided by the amount of O_2 consumed (E). Error bars show SEM, each dot represents an individual animal. *P<0.05, calculated by student t-test.

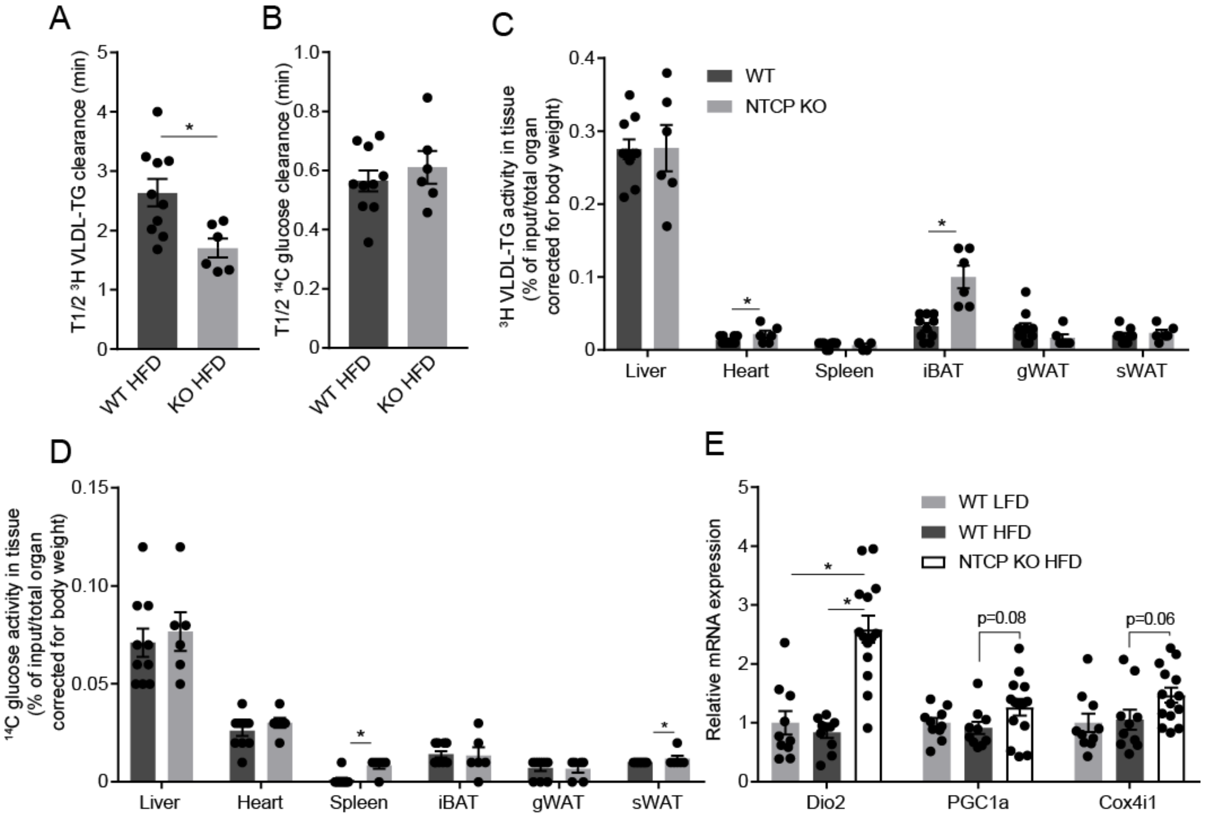

Figure S5. BAT thermogenesis in NTCP KO mice after prolonged bile acid signaling. (A-D) 3-week high fat diet (HFD)-fed WT (*n* = 10) and Na^+-taurocholate co-transporting polypeptide (NTCP) KO mice (*n* = 6) were 4-5 hours fasted and subsequently i.v. injected with radiolabeled [^{14}C]deoxyglucose and [^{3}H]triolein-labeled VLDL-like particles. Plasma clearance and uptake by organs at 15 minutes after injection were determined by assessing ^{3}H and ^{14}C-activity by liquid scintillation counting. Blood volume was estimated as 4.706% of total body weight. Half-life ($T_{1/2}$) of ^{3}H-VLDL-triglycerides (A) and ^{14}C-deoxyglucose (B) from plasma. (C-D) Uptake of ^{3}H-VLDL-triglycerides (C) and ^{14}C-deoxyglucose (D) by organs, 15 minutes after the i.v. injection with radiolabeled particles. Data was corrected for total organ and body weight. (E) mRNA expression levels, determined by reverse transcription quantitative PCR (RT-qPCR), of Deiodinase 2 (*Dio2*), Peroxisome proliferator-activated receptor gamma coactivator 1-alpha (*PGC1A*), and Cytochrome c oxidase subunit 4 isoform 1 (*Cox4i1*) in brown adipose tissue (BAT) of WT and NTCP KO mice, fed a low fat diet (LFD) or HFD for 16 weeks (*n* = 10-13 per group). Samples are relative to the geometric mean of control genes *36b4* and *hprt* and were normalized to the WT LFD group. Error bars show SEM, each dot represents an individual animal. *P<0.05, calculated by student-t-test (A-D) or 1-way ANOVA with Tukey's multiple comparison (E).

Supplementary Table S1. Primer sequences. Primer sequences used for reverse transcription quantitative PCR (RT-qPCR) analysis of uncoupling and thermogenesis in human Simpson-Golabi-Behmel syndrome (SGBS) cells, mouse brown adipocytes, and mouse liver.

**Human primers**		
**Gene**	**Forward primer (5'-3')**	**Reverse primer (5'-3')**
Bactin	GAGCACAGAGCCTCGCCTTT	TCATCATCCATGGTGAGCTGG
HPRT	TGACCTTGATTTATTTTGCATACC	CGAGCAAGACGTTCAGTCCT
UCP1	AGGATCGGCCTCTACGACAC	GCCCAATGAATACTGCCACTC
**Mouse primers**		
36B4	CCAGCGAGGCCACACTGCTG	ACACTGGCCACGTTGCGGAC
HPRT	TTGCTCGAGATGTCATGAAGGA	AGCAGGTCAGCAAAGAACTTATAG
UCP1	CAGCTTTGCCTCACTCAGGA	AAGCATTGTAGGTCCCCGTG
Dio2	CTTCCTGGCGCTCTATGACTC	CCCCATCAGCGGTCTTCTC
PGC1A	GACTGCGGTTGTGTATGGGA	GACTGCGGTTGTGTATGGGA
Cox4i1	CCGTCTTGGTCTTCCGGTTG	ACACTCCCATGTGCTCGAAG
